# Analysis of Knowledge of Smoking-Related Diseases in Spanish Nursing Students

**DOI:** 10.3390/healthcare11101438

**Published:** 2023-05-15

**Authors:** Mario García-Suárez, Daniel Fernández-García, Beatriz Ordás-Campos, Jesús Antonio Fernández-Fernández, Carlos Méndez-Martínez, Leticia Sánchez-Valdeón, Inés Casado-Verdejo

**Affiliations:** 1 Health Research Nursing Group (GREIS), University of Leon, 24071 Leon, Spain; 2 University Hospital of Leon, 24071 Leon, Spain; 3 Department of Nursing and Physiotherapy, University of Leon, 24071 Leon, Spain; 4 Department of Nursing and Physiotherapy, University of Leon, 24401 Ponferrada, Spain

**Keywords:** tobacco, knowledge, nursing students, education, prevalence

## Abstract

Smoking causes significant morbidity and mortality worldwide, mainly in developed countries. In addition, it is the cause of numerous diseases in the body, despite the fact that the prevalence of tobacco use is decreasing. Nursing students, as future professionals, should be aware of action plans for cessation and information designed for smokers. To determine the level of knowledge among nursing students about smoking-related diseases and analyze the prevalence of student who smoke at the University of Leon, Spain, a descriptive cross-sectional study was carried out in which students were given an anonymous questionnaire, which was previously validated, during the 2021–2022 academic year. In a sample of 477 (79.5%) nursing students, a smoking prevalence of 17.6% was obtained. In addition, students’ knowledge about the diseases directly caused by tobacco consumption and others associated with exposure to environmental smoke was assessed, and in both cases (8.03 points of 9 for consumption and 5.24 of 6 to exposure), scores were obtained that allow us to state that students do not know for sure the types of diseases that are related to tobacco use and passive smoking. In spite of this, it is necessary to continue to reduce the prevalence of smoking through different programs implemented in schools and universities, as it is also necessary to improve teaching plans when explaining smoking-related diseases, so that students, in the future, will be able to advise patients correctly.

## 1. Introduction

The tobacco epidemic is one of the greatest public health threats the world has ever faced. Smoking generates a high rate of preventable morbidity and premature death. It is associated with more than 25 diseases, including cancer, cardiovascular disease, diabetes, chronic obstructive pulmonary disease, and pneumonia [1,2,3].

In 2021, the World Health Organization (WHO) stated that tobacco use kills more than 8.5 million people a year, of which more than 7 million are as a result of direct consumption. About 1.2 million are associated with non-smokers exposed to environmental smoke, and this is a large increase in cases compared to 2013 data, especially regarding the tobacco-smoke-related deaths, which have doubled [4,5].

In Spain, according to the National Institute of Statistics (INE), through the 2020 European Health Survey, the percentage of daily smokers was 16.4% of women and 23.3% of men, with the latter being the most prevalent group in all age ranges. A decrease in the smoking population has been observed, mainly between 15 and 64 years of age, confirming a decreasing trend since 2009. Regarding exposure to tobacco smoke, 87.5% of women and 85.3% of men have never or almost never been exposed to tobacco smoke indoors, but 10.2% of men and 9.4% of women between 15 and 24 years of age are in environments exposed to tobacco smoke every day [6,7].

Despite the decreasing trend in Spain, tobacco use remains a very prevalent risk factor in society. In addition, there has also been a significant increase in the use of other tobacco products, such as e-cigarettes, mainly among young people [8].

Health professionals are in a strategic position to intervene at different levels to promote healthy habits and prevent high-risk behaviors [9]. Therefore, nursing students play an important role in monitoring tobacco use due to the knowledge acquired during their academic training, which, in the future, would allow them to develop health-promotion activities, including those aimed at smoking cessation. In addition, brief smoking-cessation interventions implemented by nurses have proven to be more effective than those developed by physicians [8,10].

However, the global mean prevalence of current smoking among nursing students is 26.6% according to the review by Zeng et al., with smoking being more prevalent among males and also increasing over the academic years [11]. In addition, it has been shown that students of Health Sciences who are smokers downplay the effects of direct (consumption) and indirect (passive smoking) tobacco use [3].

Therefore, it is necessary to know the behavior of nursing students in relation to tobacco use, its derivatives, and their degree of addiction. Moreover, knowing whether they are capable of carrying out health education strategies based on their knowledge once they finish their university studies is made essential.

The aim of this study was to determine the level of knowledge about smoking-related diseases and the prevalence of smoke among nursing students at the University of Leon, Spain.

## 2. Methods

### 2.1. Design

This was a descriptive cross-sectional study. The Strengthening the Reporting of Observational Studies in Epidemiology (STROBE) guidelines [12] were adhered to throughout the study. The study was carried out at the Health Sciences School of the University of Leon, Spain. The population on which this intervention was carried out included students enrolled on the Nursing Degree Course at this University during the 2021–2022 academic year.

### 2.2. Participants

The data collection was carried out between March and June 2022. The researchers delivered the questionnaires during the class hours of the nursing students. A total of 477 students decided to participate voluntarily and met the inclusion criteria of being enrolled in one of the four years of the Nursing Degree Course. They also had to submit informed consent, together with the questionnaire, once finished.

### 2.3. Assessed Variables

The questionnaire was developed in accordance with the recommendations of the European Regional Office of the World Health Organization (2002) [13] and previously validated in a study in 2008 [14]. Data were collected by means of this anonymous questionnaire that was given by the teachers to the students.

The sociodemographic variables collected included sex, age, university campus, academic year, and previous studies. Previous studies were included for those students who entered the nursing degree from the high schools (upper secondary studies) and those who entered through other previous qualifications or exams.

Data were also collected on whether participants were smokers if they reported using tobacco and/or e-cigarettes, being former smokers, or having used tobacco and/or e-cigarettes in the past but not currently, or non-smokers if participants reported never having used tobacco or e-cigarettes. Participants who identified themselves as smokers were asked about the age at which they had their first cigarette, how long they had been using tobacco regularly, and the main reason why they started consuming. In addition, they were also asked to take the test for nicotine dependence (Fagerström test) and the test for current motivation to quit smoking (Richmond test).

In relation to physical nicotine dependence, assessment was performed by using the Fagerström test [15], which is a six-item scale that evaluates the importance of nicotine addiction by assessing the time of the first cigarette of the day, the capacity to refrain from smoking in places where it is forbidden, the most difficult cigarette to give up during the day, the number of cigarettes per day, the difference in the number of cigarettes smoked between the morning and the rest of the day, and the capacity to refrain from smoking during illness. The results obtained can be categorized as low dependence (<4 points), intermediate dependence (4–6 points), or high dependence (≥7 points).

On the other hand, in terms of assessing the motivation to quit smoking, we used the Richmond test [16], which is a four-item scale that evaluates smokers’ motivation for smoking cessation by assessing the readiness to quit smoking if it could be easily done, the authenticity of the smokers’ motivation to quit smoking, the readiness to quit smoking in the upcoming couple of weeks, and the smoker’s six-month projection concerning smoking status. The scores obtained indicated low (0–5 points), moderate (6–8 points), or high (>8 points) motivation.

Finally, participants were asked about different health problems that can develop as a direct consequence of tobacco use or those that can develop due to exposure to environmental tobacco smoke, also known as ‘passive smoking’.

Following the studies by Fernández D. [17] and Ordás B. [18], each of the answers to these questions was assigned a number: 1 to ‘Main cause’; 2 to ‘One more cause’; 3 to ‘No relationship’; and 4 to ‘Relationship unknown’, taking into account that the diseases which appear are related to tobacco consumption or exposure to tobacco smoke. Thus, depending on the answers given by the students, 1 point was assigned to the answers corresponding to 1 and 2 (positive event), −1 point to answer 3 (negative event), and 0 points to answer 4 (neutral event).

Accordingly, the researchers decided that students were not competent in knowledge about smoking-related diseases as a direct result of tobacco use if they did not score equally or more than 7 points and 5 points in those pathologies related to exposure to tobacco smoke (passive smoking).

### 2.4. Statistical Analysis

A database was created by using Epi Info 7 software for the statistical analysis of the data obtained from the questionnaire. A bivariate analysis for continuous variables (age) was performed by employing a *t*-test if they presented a normal distribution, and if not, a Mann–Whitney U test was applied. A chi-squared test or an exact Fisher test (as appropriate) was used for the bivariate analysis of the categorical variables (sex, academic year, university campus, and previous studies). A *p*-value of <0.05 was considered statistically significant.

### 2.5. Ethical Considerations

This study was approved by the Ethics Committee of the University of Leon, with registration number ETICA-ULE-030-2022. The principles of confidentiality and the signing of informed consent were required in order for students to participate in the study. In addition, students were informed that not completing the questionnaire would not affect their academic progress.

## 3. Results

### 3.1. Sociodemographic Characteristics

A total of 477 nursing students participated in this study, i.e., a participation rate of 79.5% (477/600). Of the sample, 84.5% (403/477) were female, with a mean age of 21.6 years (±5.2), studying at the university campus of Leon (67.9%), and coming from upper secondary studies (75.5%). The remaining characteristics of the participants are shown in Table 1.

### 3.2. Tobacco Use Characteristics

In total, 17.6% of the students (84/477) reported themselves as tobacco or e-cigarette users (vapers), of which 3.6% (3/84) reported themselves as ‘vapers’ only, and 8.3% (7/84) claimed to be both smokers and vapers. On the other hand, 362 (75.9%) students reported never having smoked, and 31 (6.5%) reported being former smokers.

Men smoked more than women (20.3% vs. 17.1%), those from the Ponferrada campus did so more than those from Leon (24.2% vs. 14.5%), and those who did not come from upper secondary studies smoked more compared to those who did (23.1% vs. 15.8%). Statistical differences were only found according to campus. The prevalence of consumption decreased during their academic training, so 23.6% smoked in the first year and 12.7% in the fourth year, without this difference being statistically significant. All results are shown in Table 1.

In all, 96.4% (81/84) of the exclusive tobacco users started smoking at a mean age of 15.9 (±2.3), with regular smoking starting at 17.4 (±2.2) years of age and a mean number of years smoking of 6.19 (±6.96). The most frequent reasons for starting smoking identified by the students were ‘because all their friends smoked’ (35/81; 43.2%), followed by ‘to feel integrated’ (10/81; 12.3%). 

The students showed a low dependence on tobacco use according to the Fagerström test (1.95 ± 1.98) and a moderate motivation to quit according to the Richmond test (6 ± 2.39).

Regarding the Fagerström test, 92.6% (75/81) of the students reported smoking between 1 and 10 cigarettes a day, and 63% (51/81) smoked within the first 5 min of waking up; however, for 62/81 (76.5%) of smokers, the most satisfying cigarette was not the first one of the day. According to the Richmond test, 91.4% (74/81) of the students would like to quit smoking, but only 27.2% (22/81) have a higher interest in quitting.

No statistically significant differences were found in the Fagerström and Richmond tests when comparing mean nicotine dependence and motivation to quit between sexes, university campus, academic year, and previous studies.

### 3.3. Student Knowledge

All study participants completed a questionnaire based on their knowledge about health problems associated with tobacco use or exposure to tobacco smoke.

Thus, among the health problems that could occur in users, 92.9% (443/477), 73.2% (349), and 67.5% (322) of students identified tobacco as the main cause of lung cancer, throat cancer, and laryngeal cancer, respectively. In addition, tobacco use was identified as ‘one more cause’ of peripheral vascular disease and coronary heart disease by 71.3% (340) and 62.5% (298) of the students. Finally, 27.5% (131) were unaware of the relationship between tobacco use and the occurrence of bladder cancer. The results are shown in Table 2.

The mean score obtained by the students according to the scale proposed in the method was 8.03 points (SD = 1.45, minimum of −1 and maximum of 9). Table 3 shows a synthesis of the scores obtained according to sex, university campus, academic year, and previous studies. Statistically significant differences were found regarding the students’ previous studies (*p* = 0.002), reflected by the fact that students who did not come from upper secondary studies obtained a higher score in the questionnaire (8.38 vs. 7.91). During their academic training, the scores increased significantly (*p* < 0.001), so first-year students obtained a score of 7.37 compared to fourth-year students, who obtained a score of 8.18.

As for the pathologies that can develop in association with passive exposure to tobacco smoke, most of the students identified as ‘one more cause’ the fact of suffering from the pathologies they were asked about, with 71.9% (343/477) of them highlighting cardiovascular diseases. In addition, 21.6% (103) of the students stated that they were unaware of the relationship to low birth weight in newborns due to passive exposure to maternal smoke. The results of the questions are shown in Table 2.

The mean score obtained by the students for the knowledge about the pathologies associated with environmental exposure to tobacco smoke according to the scale proposed in the method was 5.24 (SD = 1.36, minimum of −3, and maximum of 6). A synthesis of the scores obtained according to sex, university campus, academic year, and previous studies is shown in Table 3. Statistically significant differences were found regarding sex, with women obtaining higher mean scores (5.31 vs. 4.85; *p* = 0.007), and academic years, with scores increasing from 4.79 to 5.26 (*p* < 0.001).

## 4. Discussion

In the present study, the level of knowledge about the health problems caused by tobacco use and/or exposure to environmental tobacco smoke in nursing students at a Spanish university was assessed, as well as the prevalence of tobacco use. A total of 477 students (79.5%) participated in the study. The number of nursing-student participants was higher than that from many consulted studies [3,11,19,20,21,22,23,24,25,26,27] and lower than in the sample of four studies [8,9,28,29]. 

In total, 84.5% (403) of the participants in the present study were females, and the mean age was 21.6 years. This is consistent with all the studies analyzed [3,8,10,11,19,20,21,22,23,24,25,26,28,29]; in these studies, a majority of participants were female, and the age ranges were between 20 and 23 years.

Regarding the academic year in which the students were at the time of the survey, 29.4% (140) were in their first year, with this being the most prevalent year. These data are consistent with six studies [8,11,20,27,28,29] that also carried out their respective interventions on first-year students, with this being the year with the highest number of students enrolled. This fact may be due to students abandoning the degree, changing to another one, or declining to participate in the study.

In total, 17.6% of the students reported themselves as smokers in the present study, with males smoking more than females. Only this percentage was higher than the one obtained in the studies by Rodríguez-Gázquez et al. [25] and Yiğitalp G. [27], with 16.3% and 12.3% of smokers, respectively. In the remaining studies [3,8,9,11,19,20,21,22,23,24,26,28,29], the prevalence of smoking was higher, ranging from 18.2% [19] to 42.4% [22]. In all studies, it was also evident that tobacco use was more prevalent among men than women. The prevalence obtained in this study corresponds with the data obtained in Spain in recent years, confirming the decreasing trend of consumption among the young population and of men smoking more than women.

Furthermore, the present study showed a decrease in the number of smokers as the academic years progressed, which is consistent with two studies [19,22]. The opposite occurred with the results of the review by Zeng et al. [10], in which the number of smokers increased with the passage of academic years, as is consistent with the findings of other studies [3,8,9,27], possibly due to the stress and anxiety generated by advanced studies. 

As for non-smokers, 362 students (75.9%) in the present study reported never having smoked a cigarette. This percentage was similar to the one found in three studies [20,23,25]. However, in other studies, the percentages were below 68% [3,8,11,27,28]. A total of 6.5% of the participants declared themselves former smokers, which is a much lower percentage than that from other studies [3,8,11,23,25,28], with percentages between 10 and 16.7%. 

As regards the mean age the onset of smoking, it was 15.9 years of age, similar to the values obtained in most of the studies that included this variable [3,8,9,11,27]; slightly higher than the mean age obtained by Fernández et al. [18], 14.9 years; and slightly lower than the mean age of 16.8 years obtained in the study by Saraiva et al. [26]. In any case, it is clear that the majority of young smokers started using tobacco before university. Preventive strategies should therefore be targeted at the age of the onset of drug use.

The Fagerström test was used to analyze physical nicotine dependence. The participants in the present study showed a low dependence, with a mean score of 1.95 points, lower than that obtained in the study by Yiğitalp G. [27], whose mean score was 4.7 points, i.e., an intermediate physical dependence on nicotine. In the rest of the assessed studies, similar values were obtained, which, in any case, detected low levels of dependence [3,8,9,11,19,24,26]. The low levels of dependency may be due to the fact that a majority of university students are young people who, in most cases, have started using tobacco or its derivatives relatively recently and have not been able to develop a high level of addiction, smoking on specific occasions such as when they are with their friends.

Finally, in relation to the Richmond test to measure motivation to quit smoking, the students in this study obtained a mean score of 6 points, which denotes a moderate motivation to quit smoking. There are not many studies that describe the Richmond test in their results. However, it has been seen that, in most of them, the results obtained also show a moderate motivation to quit smoking [3,9,19]. One exception was the analysis by Ordás et al. [11], in which it could be observed that the motivation to quit smoking decreased progressively over the years from 2003 to 2013. Obtaining a moderate motivation to quit smoking may be related to the low physical dependence on nicotine obtained by the participants when filling out the questionnaire. Young students with low nicotine dependence can quit smoking more easily.

### 4.1. Knowledge

When assessing the level of knowledge demonstrated by the students regarding the possible pathologies associated with tobacco consumption, the results showed that 92.9%, 73.2%, and 67.5% of students determined tobacco consumption as the main cause of lung, throat, and larynx cancer, respectively. These results are similar to those obtained by Fernández et al. [19] and Ordás et al. [3,11]. The same is true for the diagnosis of peripheral vascular disease (71.3%).

Furthermore, in the present study, 27.5% of the students were unaware of the relationship between tobacco use and the development of bladder cancer, although 51.8% related it as one more cause. These results are in agreement with the study by Ordás et al. [3,11], but they differ from the work by Fernández et al. [19], in which up to 45% of the students stated that there was no relationship between tobacco use and the development of bladder cancer.

Overall, regarding the general level of knowledge about the possible pathologies associated with tobacco use, the mean score obtained by the participants was 8.03 points. Thus, it can be concluded that students have an acceptable knowledge of the direct health consequences of tobacco consumption.

In relation to the level of knowledge of the students about the possible pathologies associated with exposure to tobacco smoke, the students emphasized in more than 98% of the cases that lung cancer and other respiratory pathologies were again mainly caused by the inhalation of tobacco smoke by third parties. This is consistent with what was described in four studies [3,11,19,22].

As regards cardiovascular diseases and the occurrence of childhood asthma, the students determined that exposure to tobacco smoke was ‘one more cause’ in 71.9% and 56% of cases, respectively. These findings are similar to those obtained by Fernández et al. [19] and Ordás et al. [3,11] and lower than Ortega-Ceballos et al.’s [22] values, which were around 92%.

The main differences appeared when analyzing the possibility of a newborn having a low birth weight. In the present study, 21.6% of the students were unaware that there was any kind of relationship, although 43.4% detected it as ‘one more cause’. Although these data are consistent with three studies [3,11,19], in the study by Ortega-Ceballos et al. [22], 99% of the students determined that exposure to tobacco smoke seriously increased the risk of a newborn having a low birth weight. For this question, the mean score obtained by the students was 5.24 points, which shows that students did not clearly identify the possible side effects of environmental tobacco smoke exposure.

In light of these results, it can be deduced that the method used for scoring the level of knowledge is adequate, as the students exceeded the established cutoff point. In spite of this, it is necessary to emphasize the need for new training methods and the application of new technologies in order to carry out interventions that provide students with more resources to improve their knowledge. Furthermore, given the low prevalence of smoking coupled with low physical dependence on nicotine and moderate motivation to quit smoking, new strategies may need to be established at the university level to reduce consumption and achieve smoking cessation during the students’ stay at university.

### 4.2. Limitations

The main limitations encountered in the development of this study were associated with the scarcity of articles on smoking-related knowledge in a uniform way. Each author used a different questionnaire with different types of questions, thus making it very difficult to establish a reliable comparison of the knowledge acquired by nursing students from the time they enter university until they finish their nursing studies.

In other studies, many authors described different levels of prevalence of smokers and non-smokers. However, they did not associate this prevalence outcome with questionnaires that assessed their knowledge. Instead, the studies focused on the different diseases that study participants may have or on different attitudes toward smoking.

Another limitation is that very few studies applied the Fagerström and Richmond tests, thus making it difficult to assess the physical dependence on nicotine and the real level of motivation to quit smoking.

## 5. Conclusions

After assessing tobacco use among nursing students at a Spanish university, 17.6% of the participants were smokers. This is in line with the downward trend that has been developing among Spanish youth in recent years.

Physical dependence on nicotine in nursing students was assessed as low, and the vast majority showed a moderate motivation to quit smoking. Thus, developing measures at the school/high school level may help to prevent young people from starting to smoke.

Regarding the level of knowledge about the pathologies associated with smoking or to exposure to environmental smoke, the students obtained acceptable results. Nevertheless, much more training would be needed so that, in their professional future, they can help the rest of the population when it comes to explaining the side effects of smoking or helping them in smoking cessation.

## Figures and Tables

**Table 1:** Participants’ sociodemographic characteristics and tobacco-use characteristics.

			n	%	*p*-Value
Sociodemographic characteristics	Sex	Female	403	84.5	
		Male	74	15.5	
	University campus	Leon	324	67.9	
		Ponferrada	153	32.1	
	Academic year	First	140	29.4	
		Second	121	25.4	
		Third	114	23.9	
		Fourth	102	21.4	
	Previous studies (1)	Upper secondary	360	75.5	
		Middle grade	88	18.5	
		Univ. entrance exam for over 25 years of age	23	4.8	
		Bachelor’s degree *	4	0.9	
		Upper bachelor’s	2	0.3	
	Previous studies (2)	Upper secondary studies	360	75.5	
		No upper secondary studies	117	24.5	
	Age	Mean ± SD	21.6 ± 5.2		
Tobacco-use characteristics	Tobacco use	Smokers	84	17.6	
		Former smokers	31	6.5	
		No smokers	362	75.9	
	Sex	Female	69	17.1	0.510
		Male	15	20.3	
	University campus	Leon	47	14.5	0.009
		Ponferrada	37	24.2	
	Academic year	First	33	23.6	0.141
		Second	19	15.7	
		Third	19	16.7	
		Fourth	13	12.7	
	Previous studies (2)	Upper secondary studies	57	15.8	0.072
		No upper secondary studies	27	23.1	
	Fagerström Test	Mean ± SD	1.95 ± 1.98		
	Richmond Test	Mean ± SD	6 ± 2.39		

* In Spain, before 2010 there existed two different classifications of university studies according to their length: a 3-year degree or a 5-year degree. From 2010 onward, they have been unified to meet the standards of the rest of the European Union. In the table, these two categories are represented as ‘Bachelor’s degree’ for 3-year studies and as ‘Upper bachelor’s degree’ for 5-year studies according to the previous educational plan.

**Table 2:** Students’ knowledge about tobacco as a main cause and the effects of passive smoking.

		Main Cause		One More Cause		No Relationship		Relationship Unknown	
		n	%	n	%	n	%	n	%
Tobacco as a main cause in various diseases	Lung cancer	443	92.9	34	7.1	-	-	-	-
	Chronic bronchitis	241	50.5	229	48.0	1	0.2	6	1.3
	Pulmonary emphysema	210	44.0	242	50.8	2	0.4	23	4.8
	Throat cancer	349	73.2	123	25.8	2	0.4	3	0.6
	Peripheral vascular disease	84	17.6	340	71.3	9	1.9	44	9.2
	Bladder cancer	54	11.3	247	51.8	45	9.4	131	27.5
	Coronary heart disease	137	28.7	298	62.5	8	1.7	34	7.1
	Laryngeal cancer	322	67.5	148	31.0	1	0.2	6	1.3
	Oral cavity leukoplakia	232	48.6	171	35.9	4	0.8	70	14.7
Indirect effects of passive smoking on health	Lung cancer	224	47.0	245	51.4	4	0.8	4	0.8
	Respiratory diseases	227	47.6	244	51.2	4	0.8	2	0.4
	Cardiovascular diseases	78	16.3	343	71.9	20	4.2	36	7.6
	Childhood asthma	156	32.7	267	56.0	14	2.9	40	8.4
	Other respiratory problems in children (bronchitis, pneumonia, etc.)	134	28.1	293	61.5	15	3.1	35	7.3
	Low birth weight in newborns	152	31.9	207	43.4	15	3.1	103	21.6

**Table 3:** Comparison of mean scores for students’ knowledge about tobacco as a main cause and the effects of passive smoking.

			Mean	SD	Min	Max	*p*-Value
Tobacco as a main cause in various diseases	Sex	Female	8.06	1.44	−1	9	0.277
		Male	7.86	1.55	2	9	
	University campus	León	8.07	1.48	−1	9	0.413
		Ponferrada	7.95	1.41	3	9	
	Academic year	First	7.37	1.88	−1	9	<0.001
		Second	8.24	1.18	4	9	
		Third	8.50	0.89	5	9	
		Fourth	8.18	1.27	3	9	
	Previous studies (2)	Upper sec. studies	7.91	1.51	−1	9	0.002
		No upper sec. studies	8.39	1.19	3	9	
Indirect effects of passive smoking on health	Sex	Female	5.31	1.23	−2	6	0.007
		Male	4.85	1.90	−3	6	
	University campus	Leon	5.29	1.30	−3	6	0.460
		Ponferrada	5.17	1.48	−2	6	
	Academic year	First	4.79	1.75	−2	6	<0.001
		Second	5.37	1.08	0	6	
		Third	5.61	0.87	2	6	
		Fourth	5.26	1.34	−3	6	
	Previous studies (2)	Upper sec. studies	5.25	1.35	−3	6	0.771
		No upper sec. studies	5.21	1.38	−1	6	

## Data Availability

No additional data are available.
